# Novel nomogram for predicting pulmonary complications in patients with blunt chest trauma with rib fractures: a retrospective cohort study

**DOI:** 10.1038/s41598-023-36679-z

**Published:** 2023-06-09

**Authors:** Junepill Seok, Su Young Yoon, Jin Young Lee, Seheon Kim, Hyunmin Cho, Wu Seong Kang

**Affiliations:** 1 Department of Thoracic and Cardiovascular Surgery, Chungbuk National University Hospital, Cheongju, 28644 South Korea; 2 Department of Trauma Surgery, Chungbuk National University Hospital, Cheongju, 28644 South Korea; 3 Department of Trauma Surgery, Jeju Regional Trauma Center, Cheju Halla General Hospital, 65, Doryeong-Ro, Jeju-Si, Jeju-Do Republic of Korea

**Keywords:** Health care, Risk factors

## Abstract

The direct consequences of chest trauma may cause adverse outcomes. Therefore, the early detection of high-risk patients and appropriate interventions can improve patient outcomes. This study aimed to investigate the risk factor for overall pulmonary complications in patients with blunt traumatic rib fractures. Prospectively recorded data of patients with blunt chest trauma in a level 1 trauma center between January 2019 and October 2022 were retrospectively analyzed. The primary outcomes were one or more pulmonary complications. To minimize the overfitting of the prediction model, we used the least absolute shrinkage and selection operator (LASSO) logistic regression. We input selected features using LASSO regression into the multivariable logistic regression model (MLR). We also constructed a nomogram to calculate approximate individual probability. Altogether, 542 patients were included. The LASSO regression model identified age, injury severity score (ISS), and flail motion of the chest wall as significant risk factors. In the MLR analysis, age (adjusted OR [aOR] 1.06; 95% confidence interval [CI] 1.03–1.08; p < 0.001), ISS (aOR 1.10; 95% CI 1.05–1.16; p < 0.001), and flail motion (aOR 8.82; 95% CI 4.13–18.83; p < 0.001) were significant. An MLR-based nomogram predicted the individual risk, and the area under the receiver operating characteristic curve was 0.826. We suggest a novel nomogram with good performance for predicting adverse pulmonary outcomes. The flail motion of the chest wall may be the most significant risk factor for pulmonary complications.

## Introduction

Direct consequences of chest trauma account for approximately 25% of trauma-related mortality^1,2^. Among patients with blunt trauma, 33% have thoracic injuries, which may be fatal because they are frequently accompanied by critical conditions such as airway obstruction, loss of oxygenation, exsanguination, cardiac failure, and cardiac tamponade^1^. However, these various and complex injury patterns are often ambiguous, thus confusing clinicians. Therefore, the early detection of high-risk patients and appropriate interventions can improve patient outcomes. Previous studies have demonstrated several contributing factors for adverse outcomes such as flail chest, pulmonary contusion (PC), or traumatic brain injury (TBI)^3–9^.

As one of the most severe types of chest injuries, flail chest may be closely related to adverse outcomes^3–5^. However, the role of the flail chest per se remains controversial. The commonly used term “flail chest” is a vague and collective definition. Flail chest is classified as physiologic, diagnosed based on clinical findings in patients with the paradoxical motion of a chest wall segment; or anatomical (or flail segment), diagnosed by identifying three or more consecutive segmental rib fractures via radiographic examinations such as computed tomography (CT)^10^. Considering that some patients with anatomical flail segments demonstrate normal, symmetrical chest wall motion, or a delayed type of paradoxical chest wall motion, flail chest should be distinguished according to those two types^11–14^. However, the definitions varied among previous studies, and some did not differentiate between the flail motion and flail segment^15^.

To date, no study has clearly distinguished patients with anatomical flail chest into groups with and without paradoxical chest wall motion. Previous studies have reported that the prognosis is associated with the degree of PC^6–8^, and that the severity of rib fracture and PC were positively correlated^16,17^. Therefore, these two variables may confound each other, and distinguishing which of the two has the worse effect is challenging. Extra-thoracic injuries also crucially affect adverse outcomes such as severe TBI^9^. Other injuries in the abdomen, pelvis, or extremities that require surgery or intensive care may cause hemodynamic instability in patients. Because patients with blunt chest trauma frequently have accompanying multiple extra-thoracic injuries in real-world practice, extra-thoracic injuries can confound the study results when evaluating the role of chest wall injury in patients with blunt chest trauma.

This study investigated the risk factors for predicting pulmonary complications in patients with blunt chest trauma. We also hypothesized that paradoxical chest wall movement may be an independent risk factor of adverse outcomes and aimed to construct the prediction model for individual risk. To avoid substantial confounding, we excluded severe extra-thoracic injuries.

## Material and methods

### Study design and data source

This retrospective observational single-center study was conducted at the level 1 trauma center of the Chungbuk National University Hospital, Cheongju, Korea. All methods were carried out in accordance with relevant guidelines and regulations^18^. This study aimed to evaluate risk factors for pulmonary complications in patients with multiple rib fractures. The institutional review board (IRB) approved this study (IRB no. CBNUH 2022-11-007-001). The waiver for informed consent is approved by IRB of the Chungbuk National University Hospital.

We have prospectively recorded the data of all patients with blunt chest trauma from the time of admission, including Injury Severity Score (ISS)^19^ and Abbreviated Injury Scale (AIS)^20^. Patients’ progressions, including the presence of paradoxical chest wall motion or pneumonia during the index hospitalization, were also prospectively recorded. The patterns of rib fractures (RFX) and degree of PC were recorded once based on the initial chest CT. In our trauma center, CT scans from the head to the pelvis were performed in all the patients. CT scans of the extremities were performed if necessary. A thoracic surgeon with over 10 years of experience, affiliated with the trauma center, evaluated the thoracic parameters, with a specific focus on the rib cage and lung parenchymal parameters. Then, all findings were validated in daily multidisciplinary meetings involving a radiologist and trauma surgeons. These meetings encompassed a review of both hospitalized patients and newly admitted patients. The flail motion was also assessed by the thoracic surgeon. In cases where the thoracic surgeon was not available, we utilized the recorded video footage. The presence of flail motion was determined through the analysis of this recorded footage. If consent was not obtained or video recording was not possible, the thoracic surgeon assessed the movement of the chest wall within a span of two hospital days.

### Study population, definitions, and inclusion and exclusion criteria

This study enrolled consecutive patients with blunt chest trauma who presented to the emergency department of our trauma center between January 2019 and October 2022. We included the patients with less severe extra-thoracic injuries (head AIS, face AIS, abdomen AIS, extremities AIS, and external AIS ≤ 3) because severe extra-thoracic injuries induce severe pulmonary complications and potential bias. We excluded the patients according to the following criteria: (a) intubated initially by extra-thoracic causes such as a hemorrhagic shock, spinal cord injury, or decreased mental status associated with TBI; (b) with conditions in which the degree of PC cannot be assessed such as a totally collapsed lung due to tension pneumothorax or one lung state due to the previous history of the pneumonectomy; and (c) discharged within 24 h after presentation.

The degree of PC was scored using the blunt pulmonary contusion score (BPC18)^8,16^, which divides each lung field into upper, middle, and lower thirds. Each third received a score of 0–3 based on the density of the affected lung. According to the guidelines suggested by the Chest Wall Injury Society and former studies, we classified the degree of rib displacement into four categories as follows: grade 0, no rib fractures; grade 1, rib fractures with displacement of < 50% of rib width on axial CT; grade 2, between > 50% and < 100%; and grade 3, displacement of ≥ 100%^17,21,22^. The rib fracture location was divided into three segments by anatomic landmarks: anterior, lateral, and posterior^11,17,22,23^. However, in the cases of the upper 1st–3rd ribs and lower 9th–12th ribs, they do not follow the anatomic landmarks. In our raw database, these cases were recorded by drawing imaginary lines that coincide with the landmarks of the 4th–7th ribs. A locational classification in this way was not significantly different from those of former studies. A segmental rib fracture was diagnosed when a single rib had ≥ 2 fractures at different locations. Because the lateral segment is the most extended portion among the three segments, lateral–lateral types of segmental fractures were presented in a few patients. Flail chest was subclassified and defined as following: (a) anatomical flail segment group, patients with three or more consecutive segmental rib fractures; (b) flail motion group, patients with paradoxical movement of the chest wall during index hospitalization.

The primary outcome of our study was the overall pulmonary complications, defined as one or more of the following: (a) pneumonia, characterized by clinical signs and symptoms with quantitative microbiologic confirmation of a lower respiratory tract culture via bronchoscopy (> 10^5^ colony-forming units per mL) in ventilated patients and as ≥ 2 of purulent sputum, body temperature > 38.3 °C, leukocytosis > 11,000/dL, and aggravation of chest radiograph findings in non-ventilated patients; and (b) other complications requiring surgical treatment, such as empyema, descending aortic injury due to rib fractures, or thoracotomy due to delayed hemothorax or pneumothorax that newly occurred.

### Statistical analysis

The median and interquartile range represent continuous data, and proportions represent categorical data. Continuous data were compared using the Student’s t-test or Mann–Whitney U test. Proportions were compared using the chi-square or Fisher’s exact tests as appropriate. Significance was set at p < 0.05. All statistical analyses were conducted using the R language, version 4.1.2 (R foundation, Vienna, Austria). We used the “moonBook,” “autoReg,” “multipleROC,” “pROC,” “glmnet,” “tidyverse,” and “rms” packages for data analysis and visualization.

To minimize the overfitting of the prediction model and enhance the accuracy of the new dataset, we used the least absolute shrinkage and selection operator (LASSO) to shrink the regression coefficients to zero^24,25^. We performed a tenfold cross-validation to select an optimal hyperparameter (λ). In the cross-validation, optimal λ was selected as the most regularized model so that the error was within one standard error of the minimum^24^. We input risk factors for overall pulmonary complications into the LASSO regression model, including age, sex, body mass index, head AIS, face AIS, abdomen AIS, chest AIS, extremity AIS, external AIS, ISS, presence of the visible flail motion, BPC18, and initial ratio of arterial oxygen partial pressure to fractional inspired oxygen. The LASSO regression model also included various RFX patterns and surgical stabilization of rib fractures (SSRF).

After a feature selection using the LASSO regression model, we constructed a multivariable logistic regression (MLR) model. Based on the logistic regression model, we delineated a nomogram, a graphical calculating device that allows approximate probability computation^26^. We used a receiver operator characteristic (ROC) curve to evaluate the performance of the prediction model and calculate the area under the ROC curve (AUROC). We also compared our proposed model with conventional models including the ISS, Thorax Trauma Severity Score (TTSS)^7^, Rib Fracture Score (RFS)^27^, and chest trauma score (CTS)^28^. We used Youden’s index to calculate the optimal cut-off value^29^.

### Institutional review board statement

This study was approved by the institutional review board of the Hospital (IRB no. 2022-11-007-001). Informed consent was waived due to the study's observational nature.

## Results

### Patient characteristics

**Figure 1:**
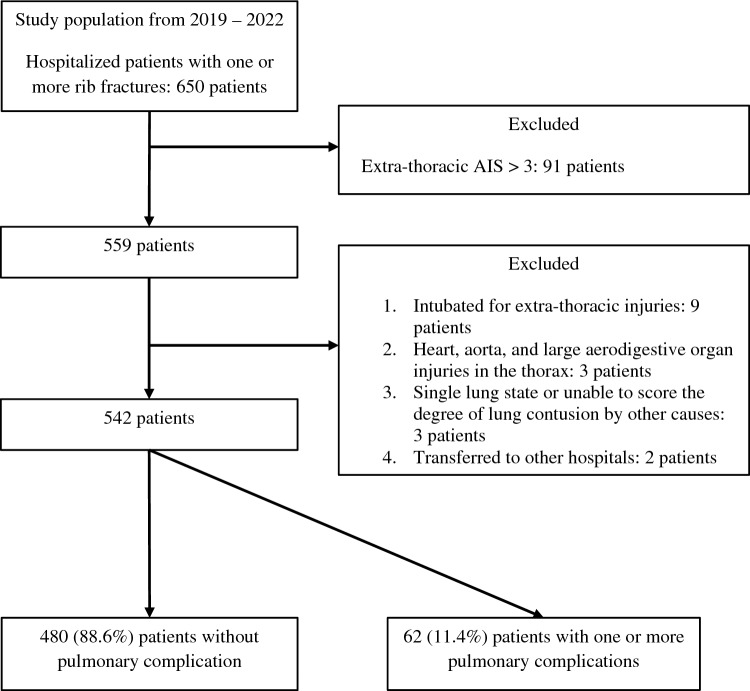
Flowchart of patient selection process.

The study population, including the inclusion and exclusion criteria, is delineated in Fig. 1. Table 1 presents the baseline characteristics and outcomes of the study population. During the study period, 650 patients with one or more rib fractures were admitted; of them, 542 were finally included. Among them, 398 (73.4%) were men. The median age was 59.0 (50.0; 71.0) years, and the median chest AIS was 3.0 (3.0; 3.0). The median number of rib fractures was 5.0 (3.0; 7.0), and 66 (12.2%) patients had bilateral rib fractures. Of the patients, 155 (28.6%) had three or more consecutive segmental rib fractures (anatomical flail segment), and 41 (7.6%) of whom exhibited paradoxical chest wall movement (flail motion).

**Table 1:** Comparison of the clinical characteristics and outcomes of patients with or without one or more pulmonary complications.

	Total (N = 542)	No complication (N = 480, 88.6%)	One or more complications (N = 62, 11.4%)	p
Sex, n (%)				0.129
Female	144 (26.6%)	133 (27.7%)	11 (17.7%)	
Male	398 (73.4%)	347 (72.3%)	51 (82.3%)	
Age, median [IQR]	59.0 [50.0;71.0]	58.0 [48.5;69.0]	70.5 [63.0;76.0]	< 0.001*
BMI, median [IQR]	23.9 [21.5;26.3]	23.9 [21.5;26.3]	23.7 [21.2;26.0]	0.495
BPC18, median [IQR]	1.0 [0.0;3.0]	1.0 [ 0.0; 2.0]	1.5 [ 0.0; 4.0]	0.012*
Initial PFR, median [IQR]	318.7 [240.9;391.7]	327.0 [250.0;407.9]	261.3 [182.5;343.8]	< 0.001*
Hospital LOS, median [IQR], day	12.0 [6.5;21.4]	10.4 [ 6.0;18.1]	22.8 [16.8;39.0]	< 0.001*
ICU LOS, median [IQR], min	0.0 [0.0;3325.0]	0.0 [ 0.0;2477.5]	8447.5 [2175.0;19,320.0]	< 0.001*
MV LOS, median [IQR], min	0.0 [0.0;0.0]	0.0 [ 0.0; 0.0]	860.0 [ 0.0;5904.0]	< 0.001*
Pneumothorax, n (%)	309 (57.0%)	255 (53.1%)	40 (64.5%)	0.119
Hemothorax, n (%)	324 (59.8%)	275 (57.3%)	38 (61.3%)	0.643
Overall pulmonary complications	62 (11.4%)			
Pneumonia	48 (8.9%)	0 (0.0%)	48 (77.4%)	
Other pulmonary complication	19 (3.5%)	0 (0.0%)	19 (30.6%)	
Rib fracture patterns				
No. of RFX, median [IQR]	5.0 [3.0; 7.0]	4.0 [3.0; 7.0]	6.0 [5.0; 9.0]	< 0.001*
≥ Grade II	2.0 [0.0; 3.0]	2.0 [0.0; 3.0]	3.0 [1.0; 5.0]	< 0.001*
No. of sRFX	0.0 [0.0; 3.0]	0.0 [0.0; 3.0]	3.0 [1.0; 4.0]	< 0.001*
Bilateral, n (%)	66 (12.2%)	54 (11.2%)	12 (19.4%)	0.103
3 or more serial RFX, n (%)	428 (79.0%)	368 (76.7%)	60 (96.8%)	< 0.001*
Anatomical flail segment, n (%)	155 (28.6%)	121 (25.2%)	34 (54.8%)	< 0.001*
Flail motion, n (%)	41 (7.6%)	19 (4.0%)	22 (35.5%)	< 0.001*
Scoring systems				
ISS, median [IQR]	14.0 [9.0; 17.0]	14.0 [9.0; 17.0]	17.5 [14.0; 22.0]	< 0.001*
AIS head	0.0 [0.0; 0.0]	0.0 [0.0; 0.0]	0.0 [0.0; 2.0]	< 0.001*
AIS face	0.0 [0.0; 0.0]	0.0 [0.0; 0.0]	0.0 [0.0; 0.0]	0.023*
AIS chest	3.0 [3.0; 3.0]	3.0 [3.0; 3.0]	3.0 [3.0; 3.0]	0.002*
AIS abdomen	0.0 [0.0; 2.0]	0.0 [0.0; 2.0]	0.0 [0.0; 2.0]	0.633
AIS extremities	0.0 [0.0; 2.0]	0.0 [0.0; 2.0]	2.0 [0.0; 2.0]	< 0.001*
AIS external	1.0 [0.0; 1.0]	1.0 [0.0; 1.0]	1.0 [0.0; 1.0]	0.078
TTSS	9.0 [7.0; 12.0]	9.0 [ 7.0;12.0]	12.0 [11.0;15.0]	< 0.001*
RFS	6.0 [4.0; 9.0]	6.0 [4.0; 9.0]	9.0 [7.0;11.0]	< 0.001*
CTS	5.0 [4.0; 7.0]	5.0 [4.0; 6.5]	6.0 [6.0; 8.0]	< 0.001*
Surgical stabilization of RFX	31 (5.7%)	19 (4.0%)	12 (19.4%)	< 0.001*

*n and No.* number, *min* minutes, *BMI* Body Mass Index, *IQR* interquartile ranges, *BPC18* Blunt Pulmonary Contusion score, *PFR* PaO_2_/FiO_2_ ratio, *ICU* intensive care unit, *MV* mechanical ventilator, *LOS* length of stay, *RFX* rib fractures, *sRFX* segmented rib fractures, *ISS* Injury Severity Score, *AIS* Abbreviated Injury Scale, *TTSS* thoracic trauma severity score, *RFS* rib fracture score, *CTS* chest trauma score.

*Statistical significance when p < 0.05.

The study population was divided into two groups as follows: with or without pulmonary complications. In total, 62 (11.4%) patients experienced one or more pulmonary complications (overall pulmonary complication group), and they were significantly older than the non-complication group (p < 0.001). The overall pulmonary complication group also demonstrated higher injury scores except for the abdomen and external AIS, more severe rib fracture patterns, and longer length of hospital stay, including stay in the intensive care unit and duration of MV dependence (p < 0.001). Of the study population, 31 (5.7%) patients underwent SSRF. Meanwhile, SSRF was performed in only 19 (4.0%) patients in the non-complication group and 12 (19.4%) patients in the overall complication group (p < 0.001).

### Risk factor analysis using the LASSO regression model

**Figure 2:**
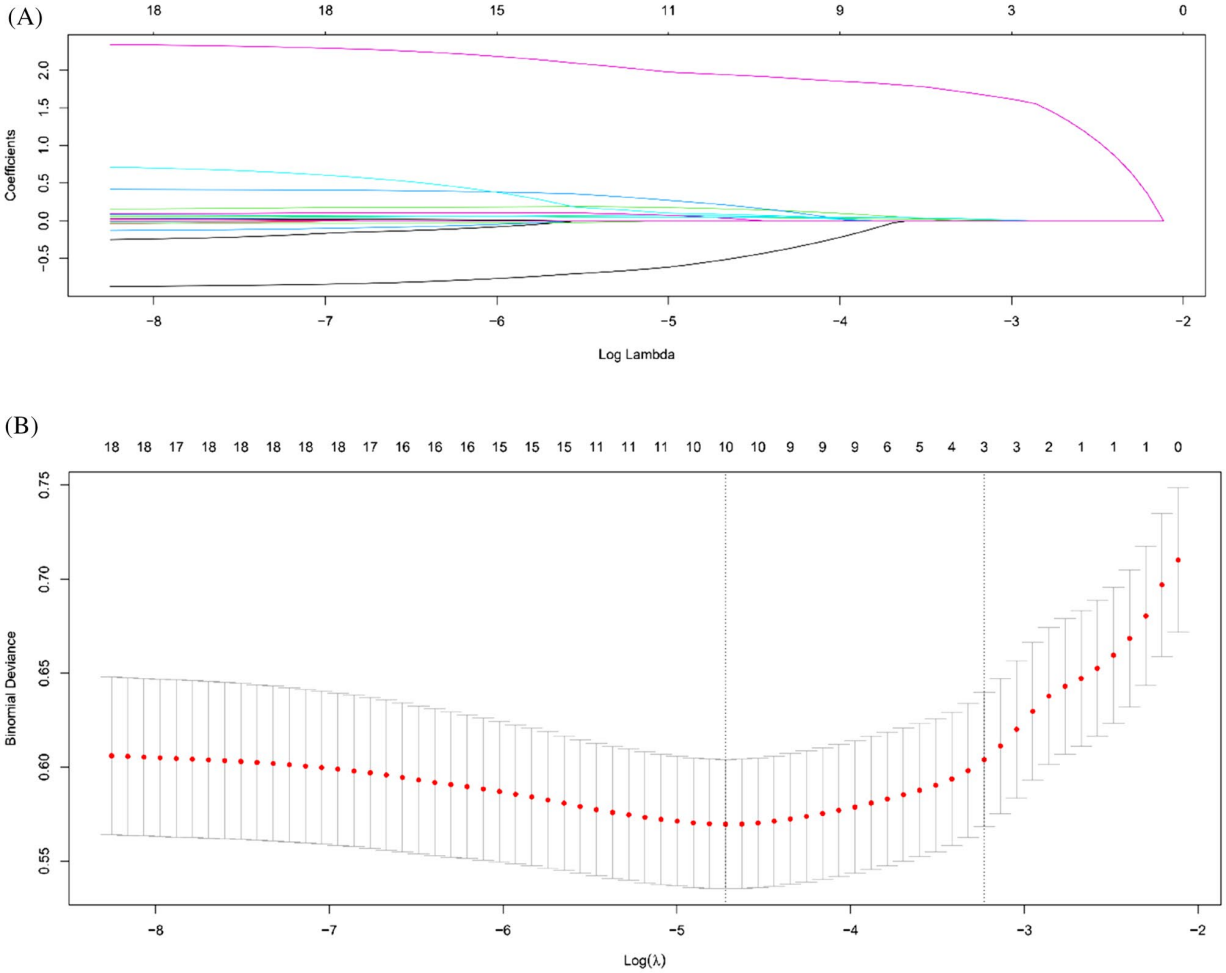
Clinical variables were selected using the least absolute shrinkage and selection operator (LASSO) logistic regression model. (**A**) Shrinkage of coefficients by hyperparameter (λ). (**B**) Hyperparameter selection (λ) using cross-validation. The dotted line indicates the value of the harmonic log (λ) when the error of the model is minimized. In the LASSO logistic regression model, three variables (age, ISS, and flail motion) were selected when log (λ) was − 3.2302.

Analysis using the LASSO logistic regression model is summarized in Fig. 2. Figure 2A delineates the shrinkage of coefficients by the hyperparameter (λ), and Fig. 2B delineates the model's accuracy via cross-validation. In the cross-validation, the optimal log (λ) was − 3.2302. At this level, three risk factors (age, ISS, and flail motion) were selected, and the LASSO shrank the coefficient estimates of the other risk factors toward zero.

### Prediction model, nomogram, and model performance

**Figure 3:**
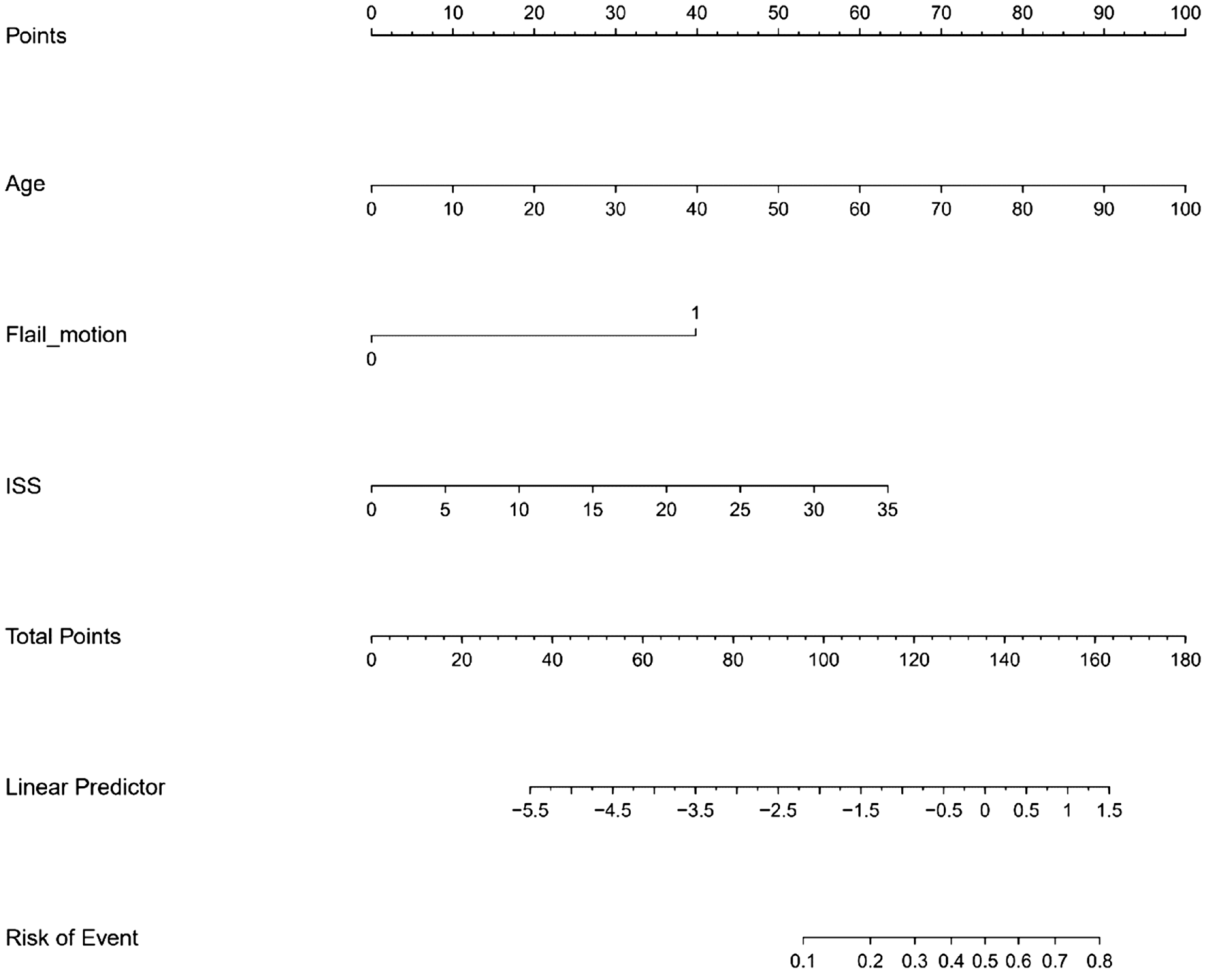
Nomogram based on multivariable logistic regression model for predicting overall pulmonary complications. A point was identified for each risk factor for overall pulmonary complications. After adding all the scores, predicted probability can be identified on the lowest rule corresponding to calculated total point.

**Figure 4:**
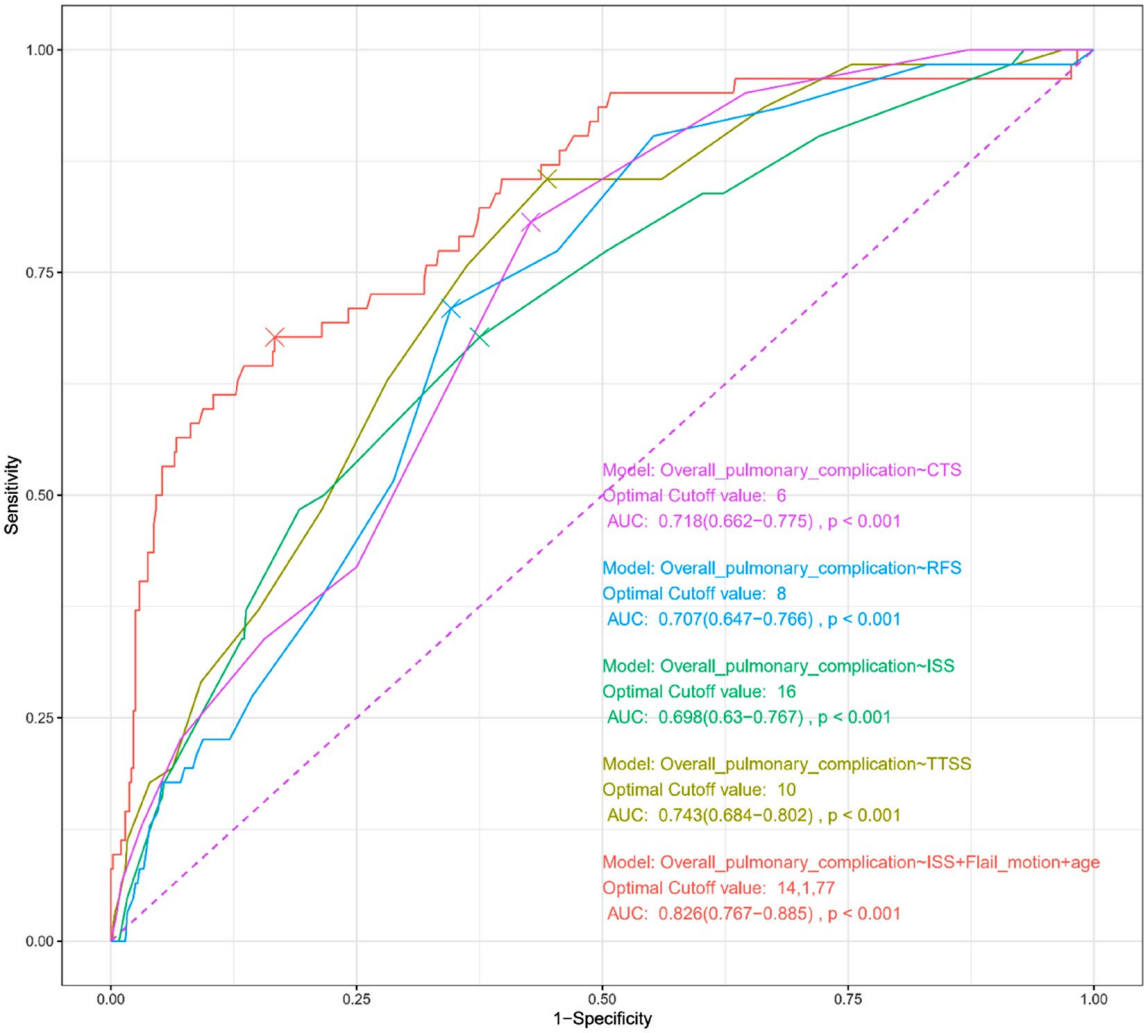
Accuracy of the multivariable logistic regression model for predicting overall pulmonary complications compared to conventional prediction models including Chest Trauma Score, Rib Fracture Score, Injury Severity Score, and Thorax Trauma Severity Score. Our proposed model yielded the highest area under the receiver operating characteristic curve.

The MLR model using three risk factors selected in the LASSO is summarized in Table 2. In the MLR analysis, age (adjusted OR [aOR] 1.06; 95% confidence interval [CI] 1.03–1.08; p < 0.001), ISS (aOR 1.10; 95% CI 1.05–1.16; p < 0.001), and flail motion (aOR 8.82; 95% CI 4.13–18.83; p < 0.001) were significant. We constructed a nomogram for predicting the personalized probability of the overall pulmonary complications (Fig. 3). Comparisons with conventional models such as ISS, CTS, RFS, and TTSS are summarized in Fig. 4 and Table 3. Our proposed model had the highest AUROC of 0.826.

**Table 2:** Multivariable logistic regression model for overall pulmonary complications.

	Univariable				Multivariable			
	cOR	95% CI of cOR		P	aOR	95% CI of aOR		P
		Lower	Upper			Lower	Upper	
Age	1.05	1.03	1.07	0.001	1.06	1.03	1.08	< 0.001
ISS	1.12	1.07	1.17	< 0.001	1.10	1.05	1.16	< 0.001
Flail motion	13.34	6.67	26.70	< 0.001	8.82	4.13	18.83	< 0.001

*cOR* crude odd ratio, *aOR* adjusted odd ratio, *CI* confidence interval, *ISS* Injury Severity Score, *AIS* Abbreviated Injury Scale.

**Table 3:** Accuracy of the prediction models.

Model	Optimal cutoff value	Sensitivity	Specificity	PPV	NPV	AUROC	95% CI of AUROC		p
							Lower	Upper	
Proposed model	Age = 77, ISS = 14, Flail motion = yes	67.7%	83.3%	34.4%	95.2%	0.826	0.767–	0.885	< 0.001
ISS	ISS = 16	67.7%	62.5%	18.9%	93.8%	0.698	0.630–	0.767	< 0.001
TTSS	TTSS = 10	85.5%	55.6%	19.9%	96.7%	0.743	0.684–	0.802	< 0.001
RFS	RFS = 8	71.0%	65.4%	21.0%	94.6%	0.707	0.647–	0.766	< 0.001
CTS	CTS = 6	80.6%	57.3%	19.6%	95.8%	0.718	0.662–	0.775	< 0.001

*PPV* positive predictive value, *NPV* negative predictive value, *AUROC* area under the receiver operating characteristic curve, *CI* confidence interval, *ISS* Injury Severity Score, *AIS* Abbreviated Injury Scale, *TTSS* thoracic trauma severity score, *RFS* rib fracture score, *CTS* chest trauma score.

## Discussion

Our study presents a prediction model for pulmonary complications in patients with blunt trauma with rib fractures. We also suggested a novel nomogram for predicting pulmonary complications. Visible flail motion of the chest wall has the most significant effect size compared to other anatomical variables in our nomogram. This result may be reasonable because the physiologic, “visible” flail motion represents the most severe form of chest injury and requires an anatomical flail segment by multiple rib fractures as a *sine qua non*^15^.

Our proposed model also has several advantages in clinical practice. First, we demonstrate a straightforward prediction model for pulmonary complications in patients with blunt chest trauma considering age, ISS, and flail motion. Because ISS and AIS have already been widely used in most trauma centers, and the paradoxical chest wall motion could be observed on the bedside, our model has good accessibility and could be easily applied in actual clinical situations. A practical protocol could be established to facilitate the decision-making process regarding admission to the intensive care unit and the implementation of close monitoring for patients with high probability as determined by the nomogram prediction model. Second, our model outperformed conventional models including ISS, TTSS, RFS, and CTS for predicting pulmonary complications in patients with blunt chest trauma. In a previous study comparing various chest trauma scoring systems in patients with isolated blunt chest trauma^8^, TTSS demonstrated the best predictive power (0.723 AUROC) for adverse pulmonary outcomes. Herein, our study also validated the predictive power of TTSS and obtained a similar result (0.743 AUROC). However, our proposed model’s predictive power (0.826 AUROC) is superior to the former scoring systems. This difference might be due to the physiologic status of patients (visible flail motion of the chest wall and anatomical components) being included in our model. Third, our nomogram would provide personalized probability and guidance for differentiated treatment strategy. The prediction of an individual risk could help clinicians determine the early intervention or close observation. Finally, the flail motion demonstrated the most significant effect size among the variables in our analysis. This may provide meaningful insight for trauma surgeons. To the best of our knowledge, this is the first study demonstrating that visible flail motion is the most significant risk factor for predicting adverse respiratory outcomes.

In contrast to our study results, other studies have already indicated respiratory failure as being more likely associated with the underlying lung injury rather than chest wall motion mechanics^15,30–32^, since Trinkle et al.’s study 50 years ago^33^. In this prospective study conducted with 30 patients with flail chest, the authors concluded that the role of flail motion was minor. However, this study has several limitations by current standards. In this era, since chest CT was not widely used, the degree of PC was solely evaluated by chest X-rays or clinical findings such as Lloyd’s criteria^34^. Moreover, accompanying extra-thoracic injuries were not classified in detail. In 1995, a canine model experiment was conducted to evaluate the effects of the artificially-created flail chests within a few hours after cutting down the ribs and also concluded that flail motion is not problematic^30^. However, in clinical situations, not all flail chests—precisely, a flail segment of the chest wall composed of segmental rib fractures—initially exhibit paradoxical chest wall motions. Respiratory failure by muscle fatigue can even occur several days after the initial trauma^11–14^. Several recent studies have also concluded that the presence of the flail chest does not affect adverse outcomes^15,32^. However, these two studies were conducted without distinction between the anatomic flail segment and the paradoxical chest wall movement. Moreover, they divided patient groups with or without PC, without considering the degree of PC.

In our study, we have recorded all patient’s data prospectively so that we could find patients with delayed-type flail chest motion. We also applied the chest CT-based BPC18 scoring system to measure the lung parenchymal injury more precisely. As result, our study identified flail motion per se as the most powerful predictor for pulmonary complications. Thus, our results may provide new insight to clinicians.

Patients’ age and ISS were also significant in our MLR model. We excluded extra-thoracic injuries with an AIS of > 3. Even in this cohort, the ISS was significantly associated with adverse outcomes, implying that extra-thoracic injuries are also crucial for predicting adverse respiratory outcomes. In a previous study, a combination of severe TBI (head AIS ≥ 3) and chest trauma was an independent predictor of pneumonia^9^. In our study, less severe TBI (head AIS < 4) was not selected in LASSO regression. Hence, further studies are warranted.

Our study has several limitations. First, the retrospective design may induce selection bias. Second, small number of patients who underwent SSRF were included. Surgical fixation might affect the patient’s prognosis. However, our principle of SSRF is very conservative, and we have performed SSRF only on the respiratory compromised patients with visible flail motion or with “on the way out” procedure after thoracotomy for another indication. We also performed SSRF on the patients with delayed-onset flail motion, although these patients had already demonstrated increase in sputum and breathing difficulty preoperatively. Hence, the probability that SSRF might lower the incidence of flail motion would be less likely in our study. Therefore, further studies investigating the SSRF’s role for preventing flail motion of the chest wall are necessary. Third, cases of severe lung trauma such as tension pneumothorax or massive hemothorax were excluded, which can induce potential bias. Fourth, this was a single cohort study, and external validation was not conducted. Multicenter trials and external validation are also warranted. Fifth, the severity of the pneumoniae as the primary outcome may vary. However, within our cohort, all patients with pneumoniae received antibiotic treatment and exhibited a higher likelihood of longer hospital and ICU stay, prolonged machinal ventilation, and higher incidence of tracheostomy compared to patients without pneumoniae. We hypothesize that every type of pneumoniae represents adverse outcomes and could potentially serve as precursors to ARDS or multiorgan failure. For future studies, a more stratified analysis is warranted. Finally, we excluded cases of severe extra-thoracic injury as they could potentially contribute to adverse pulmonary outcomes. While a more comprehensive model that includes severe extra-thoracic injuries would be desirable, it would pose significant challenges and complexities. In future studies, we intend to investigate a more comprehensive model that incorporates severe brain or abdominal injuries.

## Conclusion

The novel nomogram exhibited good performance for predicting adverse respiratory outcomes in patients with blunt chest trauma with one or more rib fractures. Our proposed nomogram may provide a tailored treatment strategy. Flail motion of the chest wall represents the most severe type of injury and the most significant risk factor. Further well-designed prospective studies to address the identified issues in this study are warranted.

## Data Availability

The datasets used and/or analysed during the current study available from the corresponding author on reasonable request.

## Abbreviations

PC: Pulmonary contusion
CT: Computed tomography
ISS: Injury Severity Score
AIS: Abbreviated Injury Scale
TBI: Traumatic brain injury
RFX: Patterns of rib fractures
BPC18: Blunt Pulmonary Contusion Score
MV: Mechanical ventilation
LASSO: Least absolute shrinkage and selection operator
MLR: Multivariable logistic regression
ROC: Receiver operator characteristic
AUROC: Area under the ROC curve
TTSS: Thorax Trauma Severity Score
RFS: Rib Fracture Score
CTS: Chest Trauma Score
SSRF: Surgical stabilization of rib fractures
